# The young scientist's guide to win the award for best presentation

**DOI:** 10.1002/cre2.47

**Published:** 2016-11-29

**Authors:** Asbjorn Jokstad

**Affiliations:** ^1^ Institute of Clinical Dentistry, Faculty of Health of Health Sciences UiT, The Arctic University of Norway Norway

I have had the pleasure to partake numerous times in judge panels in competitions for young scientists, and will encourage all senior colleagues to volunteer for such tasks. These commitments are great opportunities to learn about the latest cutting‐edge research from the select brightest and best scientists, and it is wonderful to experience their energy and enthusiasm when asked to explain about particular aspects of their ongoing research. Occasionally, judge panels fail to agree on the winner and sometimes even to select the three best presenters. The flip perspective is that the remaining finalists usually remain ignorant of why they were not shortlisted, some may be disappointed and a few even disillusioned. Please do not. This editorial may hopefully provide some guidance in preparation for your next competition. Admittedly, there are no cookbook recipes for how to proceed to win. Moreover, my advices are biased by my own academic training and experiences. Hence, other sources on the subject should also be consulted.

Your presentation starts actually with the contents of your abstract. If the instructions for abstract and subsequent oral or poster presentation specify a particular format, it is clearly unwise to deviate from the construct if the ambition is to win an award. The advice sounds obvious, but my estimate is that roughly 20% of the abstract submissions to International Association for Dental Research conferences that I have reviewed in the past for a young investigator award deviate from the instructions to authors. If you present a systematic review or outcomes of a randomized clinical trial, you should be aware of the Preferred Reporting Items for Systematic review and Meta‐Analysis (PRISMA) and Consolidated Standards of Reporting Trials guidelines (CONSORT), respectively for formatting your abstract (1).

A stated research hypothesis should be at the core of your report. Competent judges will on this basis recognize best study design to adopt, know the potential statistical variability of the measured outcomes given an adequate description of materials and methods, and hence, also the power and appropriate selection and interpretation of statistical tests. Space and time allowed, one may elaborate more or less on the basis for your hypothesis. Be mindful that judges are excited by elegant deductive logic leading up to your hypothesis and less impressed by multiple references to research that is not intimately tied to your stated hypothesis. The Ockam's razor principle should be used diligently at this stage.

Stating only a general study, aim or objective, or even leaving this out in an abstract or actual presentation begs for questions about how one decided on study sample size, expected variability of measurements, dependent on choice of apparatus, or methodology or choice of particular statistical tests. That is, subjects that perhaps are not the ones you wish to tackle on the spot from a podium. Obviously, the same questions may also be prompted from a judge panel if these details appear to be odd in context to your hypothesis. My experience is that few judges dare venturing into asking about issues in statistics. Still, it happens occasionally, and it may be wise to expect also the unexpected when presenting before a judge panel.

The fewer words that is allowed in an abstract, the more difficult it becomes to write a synopsis that contains all pertinent information and leaving out all redundant information. However, some components are essential. Any study that involves humans or animals must be backed up referring to an ethics committee approval. Even worse is to “forget” to clearly present this information up front in a poster or during a presentation. Not good. Moreover, the conclusions should always reflect your stated hypothesis and nothing less and seldom more. Often, judges are impressed by candidates who highlight the significance of their findings in terms of further research projects or possible innovations in clinical practice.

Mentors and contestant must be aware that the dominant element for picking a winner can vary slightly amongst competitions. What is essential, however, is to realize that it is the presentation and the scientific achievements of the individual contestants that are being appraised, and not the grand research projects headed by a senior P.I. to whom they are affiliated with. Candidates that come primed to embellish the achievements of their more or less sizeable research group are at a disadvantage in a competition because a judge panel will recognize the substantial number of man‐hours and most likely ask the candidate to more precisely elaborate on their actual contributions in a grander scheme. I have heard both “I did everything myself” (sic) as well as “I did the tissue analyses under supervision during my summer vacation” (sic). The candidate's response may potentially shift the focus reflexively amongst the judges from pondering whether the presenter excel as a scientist or perhaps only as a fast talker.

Finally, the actual delivery of the presentation does play a role, but the emphasis is perhaps not so much focussed on what many believe is important. Consider carefully your presentation in light of the stated criteria for the competition and consult with peers. A lively and intense presentation style may be appropriate in one setting, but perhaps, more unwelcome in another. Judges are certainly aware that some competitors arrive with a handicap because they have not had access to a mentor with the knowledge and skills to optimize their mentee's presentation. Some fellow judges argue that this is part of the awards game, and others believe that it is unfair that candidates from resourceful institutions perpetuate an “us and them” stance. I do not believe there ever will be full consensus on this issue, but I find myself tending towards the second opinion group. I had the privilege to chair the International Association for Dental Research Unilever Hatton award committee this last year, which also encompass delivering a congratulation/motivational speech to the competing finalists. The narrative below is an attempt to condense my admiration and tribute to all our young scientists who hopefully will continue their search for a better world for us all:

*Dear colleagues in research and dear friends*

*Dear IADR Unilever Hatton award finalists: I believe I speak on behalf of all my fellow judges, when I say that we truly have enjoyed the many enthusiastic presentations of your research. Please don't lose that enthusiasm.*

*The rule is that we have to select a winner and a runner‐up within the three categories. However, many of you can rightly claim that your presentation was likely at least as good and you are very right.*

*Coming from a winter country I like to think of your final as an analogy to a downhill ski race. Apologies for this who haven't tried skiing, but just try to imagine that you are plunging down an incredibly steep hill on two skis at 100 to130 kilometers per hour that include several jumps where you fly some 40‐50 meters above the ground. Yes, there is always one that takes the least amount of time from the start to the finish line. However, in Kitzbuhl in Germany this winter, which is arguably one of the most famous downhill race competitions, the result list reveals that 25 competitors finished within 1 second after the winner. Perhaps it was the poise for a moment that caused a jump that was a too long or too short, or a curve that was a few centimeters too wide, an outstretched arm to regain balance or a ski that lost some traction for a split second. However, clearly, any of these 25 competitors are capable of winning a ski race on any other given day or conditions.*

*You all plunged down a similar race on Tuesday. Obviously, you did not know your competitors. Some of you had Teflon‐coated synthetic racing dresses and ultralight kevlar skis prepared by your mentors, while some of you were on your own left with inherited leather boots fitted to wooden skis and some even carried a rucksack strapped to your back.*

*But all of you completed the race, and you did your best effort, and you should be proud. Remember that on any other given day or conditions, you could have been the selected winner.*

*I hope you will remember this IADR Unilever Hatton award final as a positive experience and that you will continue your stellar research endeavors. All of you have demonstrated that you can be a winner. So congratulations once again. We look forward listening to you in many future IADR meetings.*

Seoul, June 23, 2016

Asbjorn Jokstad, IADR Awards Committee Chair

1. Equator ‐ Enhancing the QUAlity and Transparency Of health Research. URL: http://www.equator‐network.org/

